# Use of Alcohol, Cannabinoids, Psychostimulants, and Sedatives before and during the COVID-19 Pandemic among Students in 40 European Countries

**DOI:** 10.3390/ijerph192214879

**Published:** 2022-11-12

**Authors:** Mateusz Babicki

**Affiliations:** Department of Family Medicine, Wroclaw Medical University, 51-141 Wroclaw, Poland; ma.babicki@gmail.com

**Keywords:** COVID-19, restrictions, students, lifestyle, Europe, sedatives, stimulants, alcohol, drugs

## Abstract

In March 2020, the WHO announced the COVID-19 pandemic, which has been ongoing for over 2 years. To stop the spread of the virus, the governments of many countries decided to introduce reasonable social restrictions that were suitable for pandemic waves. This led to radical changes in people’s lives, especially among students, who are very active in society. Before COVID-19, being of student age was associated with the highest frequency of stimulants use. It is important to note that drugs are taken disparately in various areas. Therefore, using the Computer-Assisted Web Interview type of study, the impact of the pandemic on the use of alcohol, cannabinoids, psychostimulants (e.g., amphetamine, methamphetamine, ecstasy) and sedatives (e.g., zolpidem, zopiclone, alprazolam, lorazepam, etc.) was assessed among students from European countries. The questionnaire included single- and multiple-answer questions. The first part concerned sociodemographic questions, while the second included questions about the use of stimulants in the last 3 months prior to participation in the study. Distribution of the survey covered the period from 31 January 2016 to 30 April 2021. A total of 17,594 European students participated in the study. The vast majority of participants were women (80.4%) and students of non-medical universities (77.2%) living in Eastern European countries (86.1%). Of all students, 15,613 (89.6%) reported alcohol drinking, 2538 (14.1%) the use of cannabinoids, 650 (3.6%) psychostimulants, and 2252 (12.5%) sedatives in the past three months. It has been shown that women are far less likely to use alcohol (OR 0.81), psychostimulants (OR 0.44) and cannabinoids (OR 0.49), while they are more likely to use sedatives (OR 1.41). During the COVID-19 pandemic, the consumption of alcohol (OR 0.55) and psychostimulants (OR 0.72) decreased and that of sleep medications increased (OR 1.17). To conclude, the COVID-19 pandemic influenced the pattern of stimulants used by students in European countries. The restriction of social interactions contributed to the decrease in the consumption of alcohol and psychostimulants but increased the use of sedatives and the frequency of their use. Women were found to use sedatives more often, while men preferred to drink alcohol and use cannabinoids or psychostimulants. It has also been shown that students of Central and Eastern Europe more often use alcohol and sedatives, while in Southern European countries psychostimulants and cannabinoids are preferred.

## 1. Introduction

Due to the rapid spread of severe acute respiratory syndrome coronavirus 2 (SARS-CoV-2), the World Health Organization (WHO) announced the coronavirus disease 2019 (COVID-19) pandemic in March 2020, which has been ongoing for over 2 years [1]. In its early stages, the governments of many countries decided to introduce reasonable social restrictions in order to stop the spread of the virus; the level of restrictions varied in each pandemic wave [2,3,4]. Restrictions included, but were not limited to, the closure of offices, shopping malls, hotels, bars and clubs. In addition, the closure of kindergartens, schools and universities forced the introduction of remote education, which, until then, had not been a popular method of education [5,6]. However, these restrictions were not identical in every European country, and as the pandemic continued, national governments pursued their own policies to counter COVID-19 [7]. This led to radical changes in people’s lives, especially among students, who are social butterflies. Previous studies have shown that in the early pandemic stages, the number of social meetings decreased sharply [8]. In addition, it has been shown that before the COVID-19 outbreak, being of student age was associated with the highest frequency of stimulants use [9,10]. Moreover, the use of, for example, sedatives—such as antidepressants, anxiolytics and hypnotic-sedatives—has increased due to pandemic-related mental distress [11]. Interestingly, the consumption aspect needs to be considered bearing in mind that sedatives are taken disparately in various areas, which generally reflects the problem with the usage of drugs overall [12,13]. The same diversity is known to be present in groups of different ages [14]. Therefore, the aim of this study was to assess the impact of the COVID-19 pandemic on the use of alcohol, cannabinoids, psychostimulants and sedatives among students from European countries. To the best of the author’s knowledge, this study is one of the few studies on such a large sample group, which covers 40 European countries and compares periods before and during the COVID-19 pandemic, which is its strong point.

## 2. Materials and Methods

This was a Computer-Assisted Web Interview (CAWI) type of study on the basis of an original proprietary questionnaire. The survey was distributed via a social network among the groups of students who were the study recipients. The participation was fully voluntary and anonymous. At each stage of the study, the respondents had the opportunity to withdraw from participation without giving a reason.

Before completing the questionnaire, the participants were informed about its goals and methodology; they then gave their informed consent to participate in the survey. Afterwards, the respondents confirmed their student status. The lack of consent and/or lack of student status confirmation resulted in the immediate termination of the study. However, if both criteria were met, the respondent continued to the appropriate part of the questionnaire. Distribution of the survey covered the period from 31/01/2016 to 30/04/2021.

The questionnaire included single-answer and multiple-answer questions. The first part concerned sociodemographic questions including age, gender, college profile (medical/non-medical) and both the year and country of study. The next part of the survey included questions about the use of alcohol, cannabinoids, psychostimulants (e.g., amphetamine, methamphetamine, ecstasy) and sedatives (e.g., zolpidem, zopiclone, alprazolam, lorazepam, etc.) in the last 3 months prior to participation in the study. Additionally, if the answer was affirmative, they were asked to specify the frequency of using stimulants: at least once a week; several times a month; not more than once a month; or once a quarter. Among students consuming alcohol, a question was also asked about the type of drink consumed (multiple-answer question). Based on the date of completing the survey, the respondents were divided into people before and during the COVID-19 pandemic. The cut-off date was 11/03/2021, i.e., the date when the pandemic was announced by the WHO. Based on EuroVoc data, the respondents were divided according to the European region of study; the following were distinguished: Western, Northern, Southern and Central-Eastern [15].

The study was conducted according to the guidelines of the Declaration of Helsinki and approved by the Bioethics Committee of the Wroclaw Medical University, Poland.

### Statistical Analysis

Statistica 13.0 (Hamburg, Germany) by StatSoft was used for the statistical analysis. The Shapiro–Wilk test was used to estimate the normal distribution. Basic descriptive statistics were employed for quantitative variables. Statistical significance between the two means was assessed using the non-parametric Mann–Whitney U test. The Chi-square (Chi^2^) test was used to determine the relationship between the compared ordinal variables.

In order to assess the risk factors for the use of individual stimulants, four complex logistic regression models were built with a backward stepwise analysis. In each case, the independent variables included age, gender, pandemic period, European region, profile and year of study. The dependent variables included the use of alcohol, cannabinoids, psychostimulants and sedatives.

## 3. Results

### 3.1. Study Group Characteristics

A total of 17,594 European students participated in the study. The vast majority of participants were women (80.4%) and students of non-medical universities (77.2%) living in Eastern European countries (86.1%). Before the outbreak of the COVID-19 pandemic, 9372 (52.2%) students completed the survey. A detailed comparison of the entire study group and the comparison with regard to the COVID-19 pandemic is presented in Table 1.

### 3.2. The Usage of Alcohol, Cannabinoids, Psychostimulants and Sedatives

Of all students, 15,613 (89.6%) reported alcohol drinking, 2538 (14.1%) the use of cannabinoids, 650 (3.6%) psychostimulants (e.g., amphetamine, methamphetamine, ecstasy), and 2252 (12.5%) sedatives (e.g., zolpidem, zopiclone, alprazolam, lorazepam, etc.) in the past three months. The most consumed drink was beer (62.8%). In response to the ongoing COVID-19 pandemic, a reduction in the percentage of people consuming alcohol was observed (90.3% vs. 83.4%; *p* < 0.001). On the other hand, the number of people drinking at least once a week increased (16.1% vs. 56.8%, *p* < 0.001). A decrease in use during the COVID-19 pandemic was also observed for the cannabinoids (14.7% vs. 14.1%; *p* < 0.05) and psychostimulants (4.2% vs. 3.0%; *p* < 0.001). However, in both cases, no statistically significant difference was observed in the frequency of use. The COVID-19 pandemic contributed to an increase in the use of sedatives (e.g., zolpidem, zopiclone, alprazolam, lorazepam, etc.) among students from 11.5% to 13.6% (*p* < 0.001). The number of students using them more than once a week also increased. A detailed summary of the use of stimulants, the frequency of their use for the entire group, and a comparison of the period before and during the COVID-19 pandemic are presented in Table 2.

### 3.3. Risk Factors for the Use of Alcohol, Cannabinoids, Psychostimulants and Sedatives

In a complex logistic regression model, it was shown that women consume much less alcohol (OR 0.81), cannabinoids (OR 0.49) and psychostimulants (OR 0.44). On the other hand, they are more likely to take sedatives than men (OR 1.41). It has been shown that the inhabitants of Eastern Europe drink alcohol (OR 1.82) and consume sedatives (OR 1.5) much more often in relation to students from Southern Europe. However, they use psychostimulants (OR 0.30) and cannabinoids (OR 0.54) much less frequently. A detailed list of factors influencing the use of stimulants is presented in Table 3.

## 4. Discussion

The aim of this study was to assess the impact of the COVID-19 pandemic on the use and frequency of alcohol, psychostimulants, cannabinoids and sedatives among students from European countries. The results of the study indicate that, of the substances analyzed, students were most likely to consume alcohol both before and during the pandemic; however, they consumed alcohol much less frequently during the initial phase of the pandemic. This may have been caused by the lockdown, which restricted social meetings. The data in the literature seem to certify this: Steffen et al. indicated that 44% of young adults reported drinking less; at the same time, only 7% of adults reported the same. On the other hand, an increase in alcohol consumption was reported by 14% of young adults and 17% of mature adults [16]. Similar observations were noted by Mangot-Sala et al., that alcohol consumption was generally lower during the pandemic than in previous years [17]. The change in drinking habits when, for example, smaller amounts were consumed more frequently with the family is in line with the observation from this research, where many students drank less but more frequently [16,18,19]. Undoubtedly, the consumption pattern depends on the age group and many other predictors, as has already been summarized [20]. The above findings also certify the observation of our study that alcohol consumption increases among students in their final years of study—these people are gradually changing from young adults to mature adults. In contrast, a study by A. Zysset et al. found that in the early stages of the pandemic, one in four students surveyed developed riskier alcohol-related behaviors [21]. Similar observations were made among American students, where alcohol consumption increased significantly after the campus shutdown [22]. Thus, the data from the literature are not conclusive, and the differences are probably due to the different period of data collection. It could also have been related to varying degrees of government restrictions and increased stress. In earlier studies, increased alcohol consumption was shown to be a common way of coping with stress [23]. 

Although no significant differences were noted for frequency, a decrease in the use of cannabinoids and psychostimulants was visible during the pandemic. This may be related to alcohol consumption, which is known to be a predictor of other drugs usage (for example nicotine). A reduced number of occasions to drink alcohol (during lockdown) could entail a small decrease in psychostimulants or cannabinoids [24]. It can also be suggested that in the early days of the pandemic, the supply chains of psychoactive drugs may have been disrupted and their availability reduced. As investigated by Gunn et al., cannabis and alcohol are co-used [25]. However, the percentage of cannabinoids use decreased only moderately, and the differences are imperceptible when comparing the whole group (not only a group before COVID-19) to those from the pandemic period. Since the study limitation is definitely the lack of addiction level measurements, it cannot be precluded that a moderate to no decrease in cannabinoids use is due to addiction [26]. 

In contrast to the reduction in alcohol, cannabinoids and psychostimulants usage during the pandemic, the consumption of sedatives increased. There are studies confirming that since the beginning of the COVID-19 outbreak, mental distress/disorders have evidently increased, explaining the elevated intake that was observed in this study [27,28]. Another possible explanation could be a change in sleep patterns during the pandemic. Previous research has shown that during a lockdown, students went to bed late, stayed up late and had numerous naps during the day. All of this leads to changes in sleep architecture, worsens the quality of sleep and may contribute to a more frequent consumption of sedatives [29].

Gender differences revealed that, compared to men, women consume more sedatives but less alcohol, cannabinoids and psychostimulants. This suits the available data reporting that men are more likely than women to use almost all types of illicit drugs and that substance usage is significantly lower for females than for males [30,31]. On the other hand, when considering prescription drugs, women are more likely to seek treatment for a misuse of central nervous system depressants (which include sedatives); therefore, being at risk for greater anxiety could entail more antidepressants prescriptions, which increases the risk of misuse and overdose [32,33,34].

Finally, the results presented that, compared to Southern Europe, the students from Eastern Europe are more likely to consume alcohol and sedatives but use fewer psychostimulants and cannabinoids, the latter also being applicable for European students from the North. Regarding alcohol consumption, it has been confirmed that the highest lifetime prevalence rates (for beer, wine and breezers) were found among Eastern European countries such as Estonia, Hungary, the Czech Republic and Lithuania [35]. When it comes to sedative medication, the Statista report presenting data from 2020 indicated that, out of 19 countries in which the defined daily dosage (DDD) per 1000 inhabitants per day was evaluated, there were six countries from Central/Eastern Europe, five countries from Northern Europe and five from Western Europe, as well as four countries from Southern Europe [36]. Although the highest DDDs were observed for European countries of the North (Sweden, Iceland, Norway) and the lowest mainly in those of the West (Netherlands, Austria, Germany), the prevalence of Central/Eastern European countries was visible in those located halfway up the list (Estonia, Czechia, Slovakia), compared to only one country of the South, i.e., Spain. As for cannabinoids, a study by Gabri et al. efficiently summarizes cannabis use among adolescents and young adults [37]. The authors recapitulated the country-specific prevalence of self-reported cannabis use, within which three countries from Southern Europe (Spain, Italy, Portugal) and two from Central/Eastern Europe (Czechia, Slovakia) were included. When comparing the percentages of cannabis consumption that refer to the period when the current study also collected data (Gabri et al. provided results up to the year 2018), the mean percentage for Southern Europe is twice as large as in Central/Eastern Europe. Although this conforms to the observations presented herein, it should be certified in the future, taking into account more lenient and stringent legislations in specific countries.

The author is aware of the limitations of this work, which undoubtedly include the methodology of data collection. Since it was disseminated via the Internet, the author cannot verify the identity of the respondents. The number of people who received information about the study and the response rate is also unknown. On the other hand, this method of collecting data made it possible to reach respondents from many European countries. In addition, young respondents in particular are more willing to participate in this type of research and show greater truthfulness [38]. In a face-to-face questionnaire, more socially acceptable answers are often observed [39]. Another limitation is the vast majority of women and inhabitants of Central and Eastern Europe, which could have had an impact on the final results. The significant predominance of women may be due to the fact that women make up the majority of students from European countries [40]. In addition, past research shows that women are more likely to participate in online surveys [41]. In contrast, the preponderance of students from East-Central European countries may be because the author of the survey is a resident of that region. This may have affected the students’ level of confidence in participating in the survey. On the other hand, it could also be due to the lower visibility of the survey information in international student groups. However, it should be noted that this study covers the period before and during the pandemic and includes nearly 17,500 students, which undoubtedly proves its strength and innovation. It is also important to mention the lack of differentiation in the type of cannabinoid intake, which may show different efficacy. 

Taken together, this study provides evidence that the COVID-19 pandemic significantly influenced the pattern of alcohol, cannabinoids, psychostimulants and sedatives used by students in European countries. The restriction of social interactions contributed to the decrease in the consumption of alcohol and psychostimulants. On the other hand, the COVID-19 pandemic has contributed to a significant increase in the use of sedatives and the frequency of their use. The logistic regression analysis showed that women use sedatives more often, while men prefer to drink alcohol and use cannabinoids or psychostimulants. It has also been shown that students of Central and Eastern Europe use alcohol and sedatives more often, while in Southern European countries psychostimulants and cannabinoids are preferred. 

Undoubtedly, the results of this study show that the use of psychoactive substances is a serious problem among students. The ongoing COVID-19 pandemic has affected their use in different ways. It is necessary to continue monitoring the phenomenon and take appropriate steps to reduce the use of stimulants, the excessive use of which can lead to addiction and serious health consequences.

## 5. Conclusions

The COVID-19 pandemic has significantly changed the patterns of psychoactive substance use by students: alcohol consumption has decreased, and the use of sedatives has increased. Women evidently use more sedatives but consume less alcohol. Among the students of Central and Eastern European countries, the most frequently used stimulant is alcohol.

## Figures and Tables

**Table 1:** Characteristics of the study group and the comparison regarding the COVID-19 pandemic.

Variable		Whole Group N (%)	Before Pandemic N (%)	During Pandemic N (%)	Chi^2^	*p*
Age M ± SD		22.13 ± 2.97	22.18 ± 2.58	22.06 ± 3.34	---	0.938
Sex	Female	14,441 (80.4)	7404 (79.0)	7037 (82.0)	25.54	**<0.001**
	Male	3513 (19.6)	1968 (21.0)	1545 (18.0)		
University profile	Medical	4089 (22.8)	2404 (36.7)	1685 (19.6)	92.21	**<0.001**
	Non-medical	13,865 (77.2)	6968 (73.3)	6897 (80.4)		
Study year	I	5170 (28.8)	2046 (21.8)	3124 (36.4)	532.64	**<0.001**
	II	3220 (18.0)	1978 (21.1)	1242 (14.5)		
	III	3401 (18.9)	2037 (21.7)	1364 (15.9)		
	IV	2705 (15.1)	1468 (15.7)	1237 (14.3)		
	V	2609 (14.5)	1420 (15.2)	1189 (13.9)		
	VI	849 (4.7)	423 (4.5)	426 (5.0)		
COVID-19 pandemic status announcement	Before pandemic	9372 (52.2)	---	---	---	---
	During pandemic	8582 (47.8)	---	---		
Part of Europe	Central and Eastern Europe	15,453 (86.1)	8061 (86.0)	7392 (86.1)	23.94	**0.003**
	Western Europe	969 (5.4)	457 (4.9)	512 (6.0)		
	Northern Europe	541 (3.0)	278 (3.0)	263 (3.1)		
	Southern Europe	881 (5.5)	576 (6.1)	415 (4.8)		

Statistically significant values are in bold.

**Table 2:** Percentage comparison of students using alcohol, psychostimulants, cannabinoids and sedatives in relation to the COVID-19 pandemic.

Variable		Whole Group N (%)	Before Pandemic N (%)	During Pandemic N (%)	Size Effect	*p*
Alcohol		15,613 (89.6)	8459 (90.3)	7154 (83.4)	188.66 ^#^	**<0.001**
Beer		11,267 (62.8)	6325 (67.5)	4942 (57.6)	187.95 ^#^	**<0.001**
Wine		10,545 (58.8)	5811 (62.0)	4734 (55.2)	86.52 ^#^	**<0.001**
Spirits		8611 (47.9)	4947 (52.8)	3664 (42.7)	182.77 ^#^	**<0.001**
Cocktails		7571 (42.2)	4205 (44.9)	3366 (39.2)	58.56 ^#^	**<0.001**
Frequency of use (N = 15,613)	At least once a week	5422 (34.7)	1361 (16.1)	4061 (56.8)	0.685 *	**<0.001**
	A few times a month	3676 (23.5)	2183 (25.8)	1493 (20.9)		
	Not more often than once a month	4747 (30.4)	4747 (56.1)	0 (0.0)		
	Once in the last 3 months	1768 (11.3)	168 (2.0)	1600 (22.4)		
Cannabinoids		2538 (14.1)	1376 (14.7)	1162 (14.1)	4.82 ^#^	**0.029**
Frequency of use (N = 2538)	At least once a week	453 (17.8)	237 (17.2)	216 (18.6)	0.047 *	0.127
	A few times a month	325 (12.8)	170 (12.3)	155 (13.3)		
	Not more often than once a month	649 (25.6)	337 (24.6)	312 (26.9)		
	Once in the 3 months	1111 (43.8)	632 (45.9)	479 (41.2)		
Psychostimulants		650 (3.6)	393 (4.2)	257 (3.0)	18.44 ^#^	**<0.001**
Frequency of use (N = 650)	At least once a week	102 (15.7)	63 (16.0)	39 (15.2)	0.034 *	0.862
	A few times a month	166 (25.5)	103 (26.2)	63 (24.5)		
	Not more often than once a month	92 (14.2)	57 (14.5)	35 (13.6)		
	Once in the 3 months	290 (44.6)	170 (43.3)	120 (46.7)		
Sedatives		2252 (12.5)	1080 (11.5)	1168 (13.6)	17.81 ^#^	**<0.001**
Frequency of use (N = 2252)	At least once a week	820 (36.4)	372 (34.3)	448 (38.4)	0.062 *	**0.034**
	A few times a month	419 (18.6)	215 (19.8)	204 (17.4)		
	Not more often than once a month	528 (23.4)	243 (22.4)	285 (24.4)		
	Once in the 3 months	485 (21.6)	254 (23.5)	231 (19.8)		

^#^ Chi^2^. * Cramér’s V. Statistically significant values are in bold.

**Table 3:** Complex logistic regression model showing the impact of age, gender, university profile, studying year and area, and the impact of COVID-19 pandemic on the consumption of alcohol, cannabinoids, psychostimulants and sedatives.

Variable		Alcohol		Cannabinoids		Psychostimulants		Sedatives	
		OR (95% Cl)	*p*	OR (95% Cl)	*p*	OR (95% Cl)	*p*	OR (95% Cl)	*p*
Age M ± SD		0.96 (0.94, 0.97)	**<0.001**	0.96 (0.94, 0.97)	**<0.001**	0.95 (0.92, 0.97)	**0.001**	1.04 (1.02, 1.05)	**<0.001**
Sex	Female	0.81 (0.72, 0.91)	**<0.001**	0.49 (0,45, 0.55)	**<0.001**	0.44 (0.37, 0.52)	**<0.001**	1.41 (1.24, 1.59)	**<0.001**
University profile	Medical	----	---	----	---	----	---	----	---
Study year	II	1.04 (0.91, 1.19)	0.498	----	---	----	---	0.85 (0.74, 0.97)	**0.004**
	III	1.23 (1.08, 1.42)	**0.002**	----	---	----	---	0.82 (0.72, 0.94)	**0.019**
	IV	1.31 (1.14, 1.52)	**<0.001**	----	---	----	---	0.77 (0.69, 0.89)	**<0.001**
	V	1.55 (1.32, 1.82)	**<0.001**	----	---	----	---	0.71 (0.61, 0.84)	**<0.001**
	VI	1.42 (1.13, 1.78)	**0.003**	----	---	----	---	0.98 (0.78, 1.23)	0.854
COVID-19 pandemic status announcement	During pandemic	0.55 (0.50, 0.60)	**<0.001**	----	---	0.72 (0.61, 0.84)	**<0.001**	1.17 (1.06, 1.28)	**<0.001**
Part of Europe	Central and Eastern Europe	1.82 (1.53, 2.17)	**<0.001**	0.54 (0.46, 0.64)	**<0.001**	0.30 (0.24, 0.39)	**<0.001**	1.50 (1.97, 1.88)	**<0.001**
	Western Europe	1.27 (0.99, 1.60)	0.050	0.84 (0.66, 1.04)	0.119	0.82 (0.59, 1.15)	0.258	1.03 (0.76, 1,40)	0.86
	Northern Europe	1.12 (0.86, 1.47)	0.404	0.72 (0.55, 0.96)	**0.026**	0.84 (0.56, 1.27)	0.421	1.05 (0.74, 1.50)	0.783

OR—odds ratio; 95% CI—95% confidence interval; statistically significant values are in bold.

## Data Availability

The data presented in this study are available on request from the corresponding author.
